# Multiple lesion-specific somatic mutations and bi-allelic loss of *ACVRL1* in a single patient with hereditary haemorrhagic telangiectasia

**DOI:** 10.1038/s41431-025-01962-2

**Published:** 2025-10-29

**Authors:** Pernille Darre Haahr, Qin Hao, Klaus Brusgaard, Martin Jakob Larsen, Bibi Lange, Annette Dam Fialla, Mikkel Seremet Kofoed, Jens Kjeldsen, Nicolai Aagaard Schultz, Anette Drøhse Kjeldsen, Pernille Mathiesen Tørring

**Affiliations:** 1 Department of Oto-rhino-laryngology, Odense University Hospital, Odense, Denmark; 2 Department of Gastroenterology, Odense University Hospital, Odense, Denmark; 3 HHT Center, Odense University Hospital, Odense, Denmark; 4 Department of Clinical Genetics, Odense University Hospital, Odense, Denmark; 5 Department of Clinical Genetics, Vejle Hospital, Vejle, Denmark; 6 VASCERN HHT Reference Center, Odense University Hospital, Odense, Denmark; 7 Department of Surgery and Transplantation, Rigshospitalet, Copenhagen University Hospital, Copenhagen, Denmark

**Keywords:** Genetics research, Chromosome abnormality

## Abstract

Hereditary Haemorrhagic Telangiectasia (HHT) is an autosomal dominant vascular disorder characterized by mucocutaneous telangiectasias and arteriovenous malformations (AVMs) in internal organs. It is mainly caused by heterozygous pathogenic variants in *ENG*, *ACVRL1* or *SMAD4*. Somatic mosaic mutations in the functional allele of HHT-causing genes have been identified in skin telangiectasias and AVMs of HHT patients, which is suspected to drive formation of telangiectasias and AVMs. Our objective was to further support and clarify the pathogenetic mechanism of HHT lesion genesis by analysing several HHT lesion biopsies; all from a single HHT patient caused by a germline deletion of the entire *ACVRL1* gene. Deep exome sequencing was performed on DNA from multiple fresh tissue biopsies from the same HHT patient; six hepatic AVM samples, two macroscopic normal hepatic control samples, and three mucocutaneous telangiectasia biopsies. Somatic mosaic lesion-specific *ACVRL1* variants were identified in four hepatic AVM samples and in one telangiectasia. Two different somatic variants (c.293A>G; p.Asn98Ser and c.1378-199C>A) were identified in several lesions from the same liver. Additionally, a third lesion-specific somatic variant (c.614T>G; p.Val205Gly) was identified in one skin telangiectasia. We identified in total 3 different somatic variants, which are expected to contribute to the pathogenesis of HHT vascular lesions. These data further support the second-hit pathophysiological mechanism to explain the multifocality of vascular lesions in HHT. This is the first report to perform deep sequencing on multiple samples from both several visceral AVMs and telangiectasias originating from one single HHT patient.

## Background

Hereditary Haemorrhagic Telangiectasia (HHT) (OMIM 187300, 600376 and 175050) is an autosomal dominant vascular disorder characterized by abnormal blood vessel formation, due to genetic mutations in the TGF-beta pathway, which regulate angiogenesis and vasculogenesis [1]. This condition leads to a predisposition for telangiectatic lesions in cutaneous and mucosal tissue, and arteriovenous malformations (AVMs) in visceral organs, including liver, lungs and brain. AVMs can cause serious complications such as hemorrhage, anemia, and hemodynamic alterations. The clinical manifestations of HHT are highly heterogeneous, even within families, and the disorder is multifocal with high, however age-dependent, penetrance.

Most HHT AVMs are believed to be congenital, whereas mucocutaneous telangiectasias increase in number over the years, predominantly in adult life [2–4].

HHT is mainly associated with germline pathogenic variants in three genes: *ENG* (Endoglin, OMIM 131195), *ACVRL1* (activin receptor-like kinase 1, OMIM 601284) and *SMAD4* (OMIM 600993), all of which play important roles in the regulation of angiogenesis and vascular integrity [5–7].

The HHT diagnosis is based on the clinical consensus ‘Curaçao Criteria’, and fulfilling at least 3 of 4 criteria are diagnostic of HHT [8]. It can be problematic to base the diagnosis only on clinical examination, due to the age-dependent nature of the disorder, hence genetic testing is often a useful part of the HHT diagnostics.

Originally, HHT was thought to follow a haploinsufficiency model, which was proposed because individuals with a single germline mutation in either *ENG* or *ACVRL1* exhibited clinical features of HHT, suggesting that the reduction in gene dosage alone could cause the disease manifestations [9–11]. However, haploinsufficiency does not comprehensively explain the clinical heterogeneity and multifocality observed in HHT patients. Therefore, for years, it has been postulated that the clinical heterogeneity and multifocality observed in HHT patients, could be due to the existence of additional triggers, including a somatic loss of the second allele, via a Knudsonian two-hit mechanism [12]. According to this model, individuals inherit a germline mutation in one allele of a disease-associated gene (first hit), predisposing them to the condition. A second, acquired genetic alteration of the wild-type copy of the gene (second hit) is required for HHT lesions to develop. The germline variant and somatic second hit in combination result in the functional impairment of the corresponding gene-encoded protein and subsequent development of AVMs. HHT would, according to this model, be dominantly inherited, but recessively expressed following a second post-zygotic somatic genetic or epigenetic event. The variability of the time of occurrence of this second event could explain the variable age of onset of the various HHT lesions. Endothelial cells are considered the target cell in HHT.

In 2019, Snellings et al. showed that biallelic loss of *ENG* or *ACVRL1*, in fact, may be required in the development of HHT telangiectasia [13]. In examining multiple dermal telangiectasias from several HHT patients, they identified low-frequency somatic mutations in the same gene as the causal germline mutation, and they showed that independent lesions from a single patient harbor lesion-specific somatic mutations. In 2024 and 2025, three studies, investigating HHT AVM samples, identified low-frequency somatic mutations in tissue from multiple AVMs from multiple HHT patients carrying germline variants in *ENG*, *ACVRL1* or *SMAD4* [14–16].

It has been suggested that, besides somatic mutations in the wildtype HHT gene allele, there could be other triggers involved in generating the vascular lesions. These triggers may be mechanical trauma, inflammation, vascular injury, angiogenic stimuli, shear stress, and modifier genes [17]. These triggers may act as secondary events in combination with the somatic second hit to provoke HHT lesions, or they may precede the somatic mutation and contribute to its induction [18].

In other multifocal vascular disorders, similar to HHT, multiple publications have demonstrated somatic second hits as part of the disease mechanism to explain the development of multifocal vascular lesions. These include somatic mutations in *RASA1* in lesions from patients with capillary malformation-arteriovenous malformation (CM-AVM) and *TIE2* (*TEK*) somatic mutations in lesions from patients with venous malformations [19, 20].

To our knowledge, this is the first report to perform deep sequencing on multiple samples from both several visceral AVMs and telangiectasias originating from one single HHT patient.

## Material and methods

### Patient

A female, age 40, with clinically and genetically diagnosed HHT, was evaluated at the HHT centre at Odense University Hospital, exhibited hepatic arteriovenous malformations (HAVM) grade 4 according to the Buscarini score [21]. Previous Multiplex ligation-dependent probe amplification (MLPA) analysis had identified an *ACVRL1* whole-gene deletion. Right sided heart catheterization showed signs of high output cardiac failure, but no pulmonary hypertension (CO 9.8 L/min, CI 5.3 L/min/m2, PAP 19 mmHg, PCWP 11 mmHg). The patient did not have pulmonary AVMs but experienced epistaxis, anaemia and iron deficiency, which was corrected with laser treatment and iron supplement. The patient underwent three treatments of Bevacizumab, however, due to continuing rise in especially ALP and ALAT, and severe side effects, the patient was listed for a liver transplant. The liver transplantation was performed without complications.

### Sample collection

**Fig. 1:**
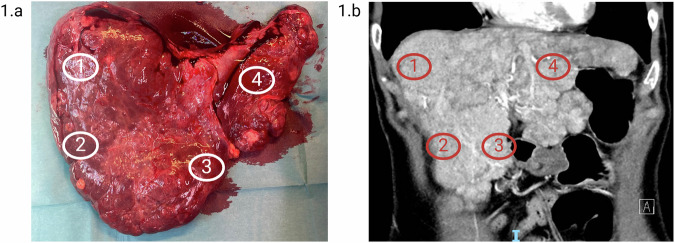
Images of liver. **a** Extracted liver displaying visible arteriovenous malformations (AVMs) and multiple focal nodular hyperplasias (FNH). **b** Coronal contrast-enhanced CT scan in the arterial phase, showing the liver before transplantation with clearly visible intrahepatic arteries and multiple FNH lesions. Biopsies were taken from marked areas: (1) HAVM in hepatic segment 7 (sample ID: G142-3 and G142-4), (2) HAVM in hepatic segment 6 (sample ID: G142-1 and G142-2), (3) HAVM in hepatic segment 5 (sample ID: G142-5 and G142-6), and (4) macroscopic normal tissue in hepatic segment 4 (sample ID: G142-N1 and G142-N2). Created with BioRender.com.

Biopsies from the hepatic vascular lesions were collected immediately after liver-extraction. A total of 8 biopsies were collected, six from objectively AVM affected areas (G142-1-6) (two biopsies each from liver segments 5, 6, and 7) and two from macroscopic normal tissue (G142-N1-2) (from liver segment 4). Segments 5 and 7 had macroscopic more severe AVM formation than segment 6. Figure 1 shows the liver after extraction and a CT scan of the liver.

Biopsies from the nasal and cutaneous telangiectasias were collected by an experienced rhinologist in local anesthesia and contained macroscopically visible telangiectasias. The biopsies were collected as close to the telangiectatic lesion as possible ensuring inclusion of the lesion in the biopsy. All AVM and mucocutaneous telangiectasia samples were immediately preserved in RNA*later* solution (ThermoFisher Scientific). An EDTA-blood sample was collected.

### DNA extraction

Blood samples: DNA was purified using the Maxwell® RSC DNA Blood DNA Kit (Promega, Sweden).

Tissue, skin and mucosal samples: DNA was purified using the Maxwell® RSC DNA Tissue DNA Kit, AS1610 (Promega, Sweden).

### Exome sequencing and Data Analysis

Sample preparation was performed using 400 ng DNA after Covaris mechanical fragmentation. Library preparation was conducted following the Twist Exome 2.0 hybridization protocol (Twist Bioscience, Inc.). Validated libraries were pooled and sequenced in paired-end mode 2 × 150 bp the on Illumina NovaSeq 6000 platform (Illumina, Inc.). The sequencing quality criteria obliged a minimum coverage of 20x in 95% of the coding regions. The mean coverage was around 1200X.

The alignment and variant calling was performed using Illumina DRAGEN Bio.IT Platform (Illumina, Inc.). Sequencing data was aligned to reference GRCh38. Variant calling was performed by using DRAGEN DNA pipeline somatic mode and tumor-normal mode. Copy number variant (CNV) analysis was performed by using VarSeq 2.5.0 (GoldenHelix, Inc.). Large somatic loss of heterozygosity was estimated by manually evaluation of the ratio value of copy number calculated by VarSeq.

VarSeq 2.5.0 was used for variant annotation and downstream filtering of the variants.

### Variant detection and evaluation

We specifically searched for variants in the *ACVRL1* gene. Clinical significance of the variants were evaluated according to ClinGen Hereditary Hemorrhagic Telangiectasia Expert Panel Specifications to the ACMG/AMPVariant Interpretation Guidelines for ACVRL1 Version 1.0.0 (https://cspec.genome.network/cspec/ui/svi/doc/GN135) [22]. The Genome Aggregation Database v.4.1.0 (GnomAD) was used to evaluate the allele frequency of the variants in the background population. For missense variants, the prediction software SIFT, Polyphen2.0, FATHMM, Mutation Taster and MutationAssessor were used. For splicing prediction SpliceSiteFinder-like, MaxEntScan and SpliceAI values were used.

To rule out involvement of other genetic variants a panel of HHT-related genes (*ENG, ACVRL1, SMAD4, RASA1, EPHB4, GDF2*) was examined.

## Results

We examined the hepatic AVM and telangiectasia samples with deep exome sequencing, focusing on mainly the *ACVRL1* gene, to investigate the presence and nature of second-hit somatic mutations in the *ACVRL1* gene. Further, a blood sample from the patient was examined with deep exome sequencing.

### Germline ACVRL1 whole-gene deletion

The germline heterozygous whole-gene deletion of *ACVRL1* was previously identified with MLPA analysis [23]. In this study, exome sequencing confirmed the deletion and showed it to be a heterozygous large deletion, approximately 130 kb, (NC_000012.12: g.(51847454_51888187)_(52014201_52014694)del), including the entire *ACVRL1* gene (pathogenic – C5) and four additional genes (*ACVR1B, ANKRD33, RNU5-574P and TAMALIN*), of which none are OMIM morbid genes. One break point locates upstream of *ANKRD33*, and the other breakpoint locates in the intron before the last coding exon in *TAMALIN*. The germline *ACVRL1* deletion was confirmed in all hepatic AVM and mucocutaneous telangiectasia samples, in the two macroscopically normal hepatic samples, and in the blood sample, consistent with the patient’s HHT diagnosis.

### Identification of somatic second-hit variants

**Fig. 2:**
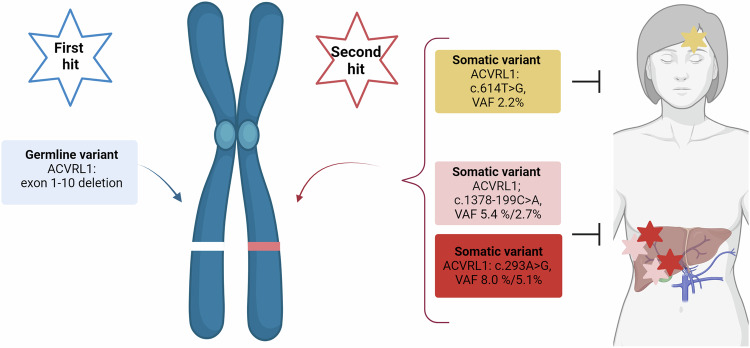
Illustrating the genetics findings; the germline variant and the multiple lesion-specific somatic variants in the liver and cutaneous samples, respectively. Created with BioRender.com.

**Fig. 3:**
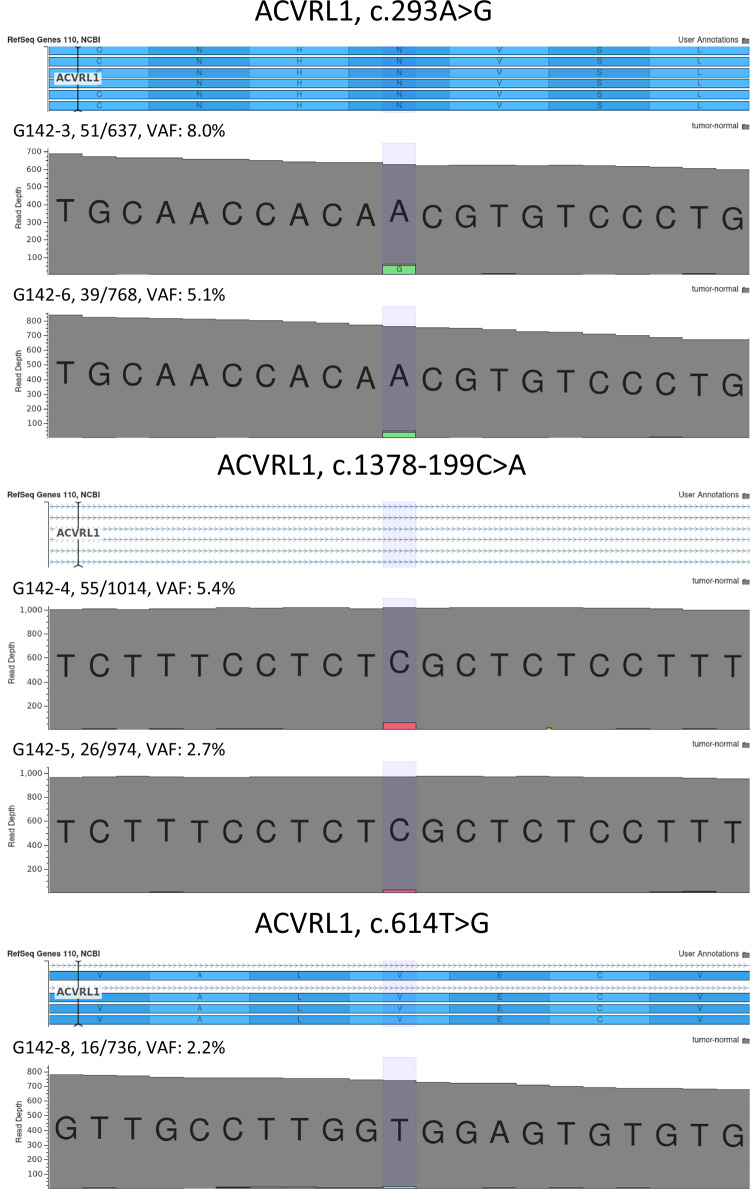
Low-frequency somatic mosaic variants in samples from an HHT patient. Varseq screen capture showing reads with somatic mosaic variants in five samples. G-142-3, G-142-6, G142-4 and G142-5 representing hepatic AVM samples and G-142-8 representing a telangiectasia.

Four out of six hepatic AVM samples and one of three mucocutaneous telangiectasias showed mosaic somatic variants in the *ACVRL1* gene (Table 1 and Fig. 2). These mutations were in *trans* with the germline deletion and not identified in the blood sample (Fig. 3).

**Table 1:** Genetic results (all samples are from the same patient). Summary of the germline and somatic genetic findings. All samples are from the same patient. ACVRL1 GenBank accession number NM_000020.3 (GRCh38).

Sample ID	Germline *ACVRL1* variant	Sample tissue	Somatic *ACVRL1* variant	Somatic variant classification	Somatic reads/total reads	Mosaicism level (VAF)	Phase
G142-1	Whole-gene deletion (130 kb)	HAVM, hepatic segment 6	None identified	-	-		-
G142-2		HAVM, hepatic segment 6	None identified	-	-		-
G142-3		HAVM, hepatic segment 7	c.293A>G, p.Asn98Ser	Likely pathogenic	51/637	8.0%	*trans*
G142-4		HAVM, hepatic segment 7	c.1378-199C>A, p?	VUS	55/1014	5.4%	*trans*
G142-5		HAVM, hepatic segment 5	c.1378-199C>A, p?	VUS	26/974	2.7%	*trans*
G142-6		HAVM, hepatic segment 5	c.293A>G, p.Asn98Ser	Likely pathogenic	39/768	5.1%	*trans*
G142-N1		Macroscopic normal, hepatic segment 4	None identified	-	-		-
G142-N2		Macroscopic normal, hepatic segment 4	None identified	-	-		-
G142-7		Telangiectasia, skin, right thigh	None identified	-	-		-
G142-8		Telangiectasia, skin, forehead	c.614T>G; p.Val205Gly	Likely pathogenic	16/736	2.2%	*trans*
G142-9		Telangiectasia, nasal mucosa	None identified	-	-		-

ACVRL1, GenBank: NM_000020.3.

*VUS* Variant of Uncertain Significance, *VAF* variant allele frequency.

Interestingly, two distinct somatic variants (*ACVRL1*, NM_000020.3: c.293A>G; p.Asn98Ser and *ACVRL1*, NM_000020.3: c.1378-199C>A) were identified in multiple lesions from different segments of the liver (Fig. 2). The somatic variant p.Asn98Ser was identified in AVM tissues in segment 5 and in segment 7 of the liver. Additionally, the somatic variant c.1378-199C>A was identified in other AVM biopsies from segments 5 and 7. A third somatic variant (*ACVRL1*; NM_000020.3: c.614T>G; p.Val205Gly) was identified in a cutaneous telangiectasia removed from the forehead of the patient (Fig. 2). Biopsy tissue details, in addition to germline and somatic variant findings are listed in Table 1.

*ACVRL1* somatic mosaicism ranged from 2.2–8.0%, with an average *ACVRL1* local total read depth of 825X. However, as one allele is deleted, due to the germline *ACVRL1* whole-gene deletion, that equals a total read depth of around 1650X. The mean total exome coverage in the biopsy samples was around 1130X.

Manual evaluation of large somatic loss of heterozygosity did not reveal any large somatic deletions in the region of *ACVRL1*.

None of the samples had pathogenic or likely pathogenic variants (C4-C5) identified using an in silico panel of additional HHT-related genes (*ENG, SMAD4, RASA1, EPHB4, GDF2*).

Finally, to rule out the possibility that the somatic variants were germline mosaic variants, we conducted deep sequencing of DNA extracted from peripheral blood DNA from the patient. All the detected somatic variant was absent from the blood. The mean total exome coverage in the blood sample was 864X.

### Classification of somatic second-hit variants

*ACVRL1*, NM_000020.3: c.293A>G; p.Asn98Ser is a heterozygous missense variant previously described as pathogenic or likely pathogenic and causing HHT in several publications [24–26]. The variant is not described in GnomAD 4.1.0, thus not observed in around 800,000 healthy individuals, and predicted damaging by 4 out of 5 prediction software. In ClinVar the variant is classified twice as pathogenic and once as a variant of uncertain significance. Based on ACMG guidelines (PS1, PM2_supporting, PP3) the variant is classified as likely pathogenic (C4).

The putative splice site variant in *ACVRL1*, NM_000020.3: c.1378-199C>A has not previously been described, in either Human Gene Mutation Database (HGMD®), GnomAD 4.1.0 or in ClinVar. The variant is predicted pathogenic in SpliceSiteFinder-like, MaxEntScan, NNSPLICE and GeneSplicer, while predicted benign in SpliceAI. Based on ACMG guidelines (PM2, PP3) the variant is classified as a variant of uncertain significance (C3). Further investigation would be required in order to reclassify the variant as likely pathogenic or pathogenic.

*ACVRL1*; NM_000020.3: c.614T>G; p.Val205Gly is a heterozygous missense variant previously described as a pathogenic variant causing HHT in several publications [27, 28]. The variant is not described in GnomAD 4.1.0 or in ClinVar and predicted damaging by 3 out of 5 prediction software. SpliceAI predicts a donor loss effect with delta score of 0.55. Based on ACMG guidelines (PS1, PM2_supporting, PP3) the variant is classified as likely pathogenic (C4).

## Discussion

In analysing several biopsies from both hepatic AVMs and cutaneous telangiectasias, all from a single HHT patient, we show independent mosaic lesion-specific somatic *ACVRL1* variants in *trans* with the germline *ACVRL1* deletion. We identified three different independent lesion-specific somatic variants, of which two were observed in multiple hepatic AVM samples. Our findings support the two-hit hypothesis in the pathogenesis of HHT-related AVMs and telangiectasias, indicating that a somatic mutation in the remaining wild-type copy of the inherited mutant gene is a necessary event in HHT lesion formation.

The identification of two distinct somatic variants in different segments of the liver highlights the heterogeneity of the somatic variant landscape and show that AVMs within the same organ can arise independently and have multiple somatic second-hit variants. Remarkably the HHT vascular lesions are both localized and tissue specific. The localized formation of vascular lesions could be explained by sporadic somatic variants generating biallelic mutations [18]. Besides somatic variants in the wildtype HHT gene allele, other triggers may contribute to vascular lesions, such as mechanical trauma, inflammation, vascular injury, angiogenic stimuli, shear stress, and modifier genes. These may act alongside or lead to the somatic second hit that provokes HHT lesions.

The identified second-hit variants were located in *trans* with the germline deletion, meaning they occur on the allele not affected by the germline deletion, leading to complete functional loss of ACVRL1 -encoded protein in the endothelial cells. It can be challenging to clearly confirm whether the identified germline and somatic variants are located in *trans* however, in this study, we can establish that conclusively as the germline variant is a whole-gene deletion. In this study the Variant Allele Frequency (VAF) was 2.2–8.0%, with the highest VAF in AVM tissue compared to the telangiectasias, possibly because visceral AVMs are larger than cutaneous telangiectasias allowing the biopsies to include a higher proportion of vascular tissue. Because the germline mutation in our study involves a complete gene deletion, the variant allele frequency of any second-hit somatic mutation reflects the full proportion of mutant cells in the sample. This contrasts with germline point mutations, where the VAF corresponds to roughly half the number of mutant cells due to the presence of one wild-type allele. Consequently, in this study we have an advantageous germline variant/deletion. Further, all biopsies inevitably include other tissue types, which will not be affected by the mutations that are located in the AVM. Further, it is well known that collection of HHT-associated lesion specimens is challenging. Biopsies are collected based on manual tissue inspection and evaluation. It is challenging to ensure a high proportion of endothelial cells in the tissue. If the endothelial cell proportion in the biopsy is low, the chance of detecting the second-hit somatic mutation decreases significantly, which also may explain the variation in VAF. In this study, we did not identify somatic second-hit in hepatic segment 6 which exhibited less severe AVM formations, possibly indicating that the tissue was less representative of the pathological state or had a lower degree of somatic mosaicism, than possible to identify with the used methodology.

Nevertheless, the VAFs we detected are relatively high, likely due to our careful collection of samples to better represent a larger proportion of relevant tissue, as these samples were specifically collected for this study. Further, our samples were fresh tissue, to avoid the bias that formalin-fixed-paraffin-embedded (FFPE) tissue would impose on genetic analysis.

Interestingly, this study identified two different somatic variants (c.293A>G: Asn98Ser and NM_000020.3: c.1378-199C>A) both observed in different liver segments (segments 5 and 7), which highlights the heterogeneity of mutational events and suggest that different AVMs within the same organ can arise independently. The presence of two postzygotic variants with differing VAFs in the same tissue suggests two individual clones with a clonal expansion process, demonstrating subclonal heterogeneity. This gives rise to thoughts concerning the timing of the occurrence of the somatic second-hit variants. In contrast to most organs, the liver is a homogenous mass, which in the embryo derives from the endoderm during gastrulation, this forms into the hepatic bud, which later, through proliferation and differentiation, develops into the mesenchymal liver components [29]. The vasculature in the liver arises in gestational week 10 deriving from the epithelium, the final vasculature is a result of a tightly controlled development, involving both angiogenesis and vasculogenesis [30]. As this study identified two different somatic variants in different areas of the liver, one can contemplate, that these variants must have happened during the development of the organ, allowing for the clonal expansion individually through the liver vasculature. It is deemed unlikely that the same somatic variant should be identified in different areas of the liver, if the variants were developed after the liver has fully developed. The occurrence of two independent somatic mutations in the liver AVM, may be partly responsible of the large size of the hepatic AVM in this patient. Moreover, the findings in this study support that hepatic AVM formation can be congenital. This is further substantiated by the previous findings of infantile intrahepatic vascular malformations [31]. The mucocutaneous telangiectasias, however, are acquired through life, increasing in numbers over the years, predominantly in adult life and in locations susceptible to sun exposure or mechanical trauma.

In our study, we detected second-hit somatic variants in the *ACVRL1* gene in 5 out of 9 samples. Two hepatic AVM samples, G142-3 and G142-6, share the same C4 variant, another two hepatic AVM samples, G142-4 and G142-5, share the same C3 variant, and one cutaneous telangiectasias sample has a different C4 variant. We did not identify second-hit somatic variants in 4 out of 9 samples; 2 hepatic AVM samples, 1 cutaneous lesion and 1 nasal mucosal telangiectasia. That is in line with previous similar studies that also did not demonstrate somatic variants in every HHT lesion studied [13–16], which could be due to tissue heterogeneity, as addressed above, or due to methodological limitations.

We used NGS exome sequencing to identify second-hit somatic variants. Exome sequencing covers the protein-coding regions of the genome and the nearby intronic regions, but it does not cover deep intronic areas, promoters, enhancers, or other regulatory regions. This could be one reason why second-hit mutations were not identified in all of the samples. Our patient has a germline heterozygous deletion of the entire *ACVRL1* gene, which complicates the detection of somatic copy number variants (CNVs). As a result, a somatic CNV may have gone undetected. The same limitation applies to somatic structural chromosome abnormalities, as the exome sequencing is not ideal for detecting those either. Somatic variant detection depends heavily on the coverage. Both sensitivity and specificity increase with coverage. The mean coverage of our exome sequencing is around 1,130X, which is quite high, but this is not always sufficient for somatic variant detection. Our method’s sensitivity is lower for variants with very low allele frequency. Lastly, variant allele frequency is also influenced by the proportion of endothelial cells in the tissue, which varies with sampling.

Beyond pathogenic CNVs/single nucleotide variants (SNVs) in the *ACVRL1* gene, other mechanisms that sequencing cannot detect may lead to gene deactivation. Somatic Loss of Heterozygosity has been reported, and furthermore, abnormal DNA methylation may promote gene silencing [15, 16, 32, 33].

## Conclusion

This is, to our knowledge, the first report to perform deep sequencing on multiple samples from both several visceral AVMs and telangiectasias originating from one single HHT patient.

Our study demonstrates mosaic, lesions-specific somatic *ACVRL1* variants in *trans* with the germline *ACVRL1* deletion across several lesion biopsies from a single HHT patient. This supports that a somatic mutation in the remaining wild-type copy of the inherited mutant gene is a necessary event in HHT lesion formation.

This study highlights the complexity of the genetic pathophysiology associated with HHT.

Understanding the mechanisms underlying AVM development in HHT has profound implications for the development of targeted therapies aimed at preventing or mitigating the vascular abnormalities characteristic of HHT.

## Data Availability

The small somatic variant calls generated from exome sequencing of the study samples are publicly available in the European Variation Archive (EVA) at EMBL-EBI under accession number PRJEB96743. The raw sequencing data generated during the current study are not made publicly available due to patient privacy, legal and ethical restrictions, in accordance with the General Data Protection Regulation (GDPR) and Danish national legislation governing the protection of personal data. The data include identifiable genetic information and thus cannot be shared publicly.
